# Use of Shenhuang paste on Shenque point improves chemotherapy induced gastrointestinal toxicity in breast cancer: A protocol for randomized controlled trial

**DOI:** 10.1097/MD.0000000000025097

**Published:** 2021-04-16

**Authors:** Rongyun Wang, Yanan Shi, Xiaohong Xie, Qinling Ge, Jingming Xu, Qiuhua Sun, Xiaohong Xu

**Affiliations:** a School of Nursing, Zhejiang Chinese Medical University; b Zhejiang Provincial Hospital of Chinese Medicine; c The First College of Clinical Medicine, Zhejiang Chinese Medical University, Hangzhou, China.

**Keywords:** breast cancer, chemotherapy, gastrointestinal toxicity, randomized controlled trial, shenhuang plaster, shenque point

## Abstract

**Background:**

Breast cancer, a malignant disorder, occurs in the epithelial tissue of the breast gland. Chemotherapy is the standard treatment for breast cancer, however, the side effect, especially gastrointestinal dysfunction, due to chemotherapy still remain major problems. Traditional Chinese Medicine has been proven therapeutically effective on reducing adverse effects caused by chemotherapy. Shenhuang Plaster

**Methods:**

The study is a randomized, placebo-controlled, blind trial. A total of 160 Chinese breast cancer patients will be enrolled and randomly allocated into the experimental group and control group in a 1:1 ratio. Patients in the experimental group will be prescribed Shenhuang plaster application on shenque point (CV8) plus chemotherapy treatment. Patients in the control group will be prescribed placebo plaster application on CV8 plus chemotherapy treatment. The acupoint application will last 3 days. The primary outcome will be the form of faces every day, and the secondary outcomes the symptom score of traditional Chinese medicine, the changes of fecal bacteria and metabolites, serum motilin, gastrin and ghrelin levels.

**Discussion:**

This study is to observe therapeutic effects with Shenhuang plaster application on CV8 to regulate chemotherapy-induced gastrointestinal toxicity in breast cancer patients.

**Trial registration:**

Chinese Clinical Trial Registry (http://www.chictr.org.cn/showproj.aspx?proj=55262) No. ChiCTR2000034313. Registered on July 2, 2020.

## 1 Introduction

Breast cancer is cancer that occurs in the epithelial tissue of the breast gland.^[1]^ In recent years, the incidence rates of female breast cancer have a slight increase and has surpassed lung cancer as the most commonly cancer worldwide.^[1,2]^ Nowadays, surgery, chemotherapy, immunotherapy and hormone, radiation and targeted therapies are acknowledged as common treatments in breast cancer.^[3–6]^ Although chemotherapy, as a commonly applied and important treatment, has significant improved overall survival in many types of cancer, it also can damage healthy cells and tissues of patients, which results in adverse outcomes that negatively affect a therapy compliance with cancer treatment.^[7]^ Gastrointestinal toxicity is a common side effect characterized by nausea, vomiting, bloating, diarrhea and constipation. It will lead to delays, adjustments, and discontinuation of treatment which is greatly impacting the quality of life in cancer patients.^[8–10]^ The incidence is about 63% to 78% in patients receiving chemotherapy.^[11]^ Furthermore, among cancer survivors, the incidence of chronic gastrointestinal toxicity, like constipation or diarrhea, has been estimated to be still as high as 49%, and the disorder would last for ten years after the treatment ended.

Complementary alternative medicine has been widely used for a long time in cancer patients. As an important part of complementary alternative medicine , Traditional Chinese Medicine (TCM) has formed its own unique system of theory, diagnosis and treatment system in Asian countries, especially in China. Currently, TCM has been increasingly used in the last decades, especially as an alternative treatment to chemotherapy, to improve clinical symptoms, relieve or reduce the adverse outcomes due to chemotherapy and prolong patients’ survival time. Acupoint application therapy is the application of Chinese herbal medicine to the corresponding points to regulating meridians, yin and yang, and qi as well as the blood of the human body. Shenhuang Plaster, consisting of *Radix et Rhizoma Rhei* (Rhubarb Root and Rhizome, Dahuang), *Radix Salviae Miltiorrhizae* (danshen root, Danshen), *Radix Ginseng* (Ginseng, Renshen), *Cortex Magnoliae Officinalis* (Officinal magnolia bark, Houpu), *Fructus Aurantii Immaturus* (Immature Orange Fruit, Zhishi), *Fructus Evodiae* (Medicinal Evodia Fruit, Wuzhuyu) and *Flos Caryophylli* (Clove, Dingxiang) is an external Chinese herbal medicine that can regulate intestinal function. Our previous studies found that Shenhuang Plaster application on Shenque point (CV8) could improve gastrointestinal symptoms after chemotherapy.^[12]^ Based on the preliminary work, this study carries out research on acupoint application for gastrointestinal side effect after chemotherapy. Through double-blind, randomized controlled study, we will verify the effect of SHP application in regulating the chemotherapy-induced gastrointestinal toxicity.

## 2 Methods

### 2.1 Registration

**Figure 1:**
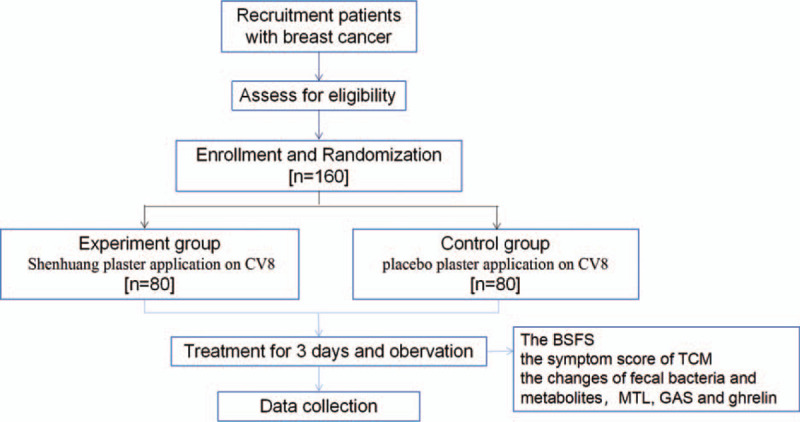
Study flowchart.

This trial had been registered before recruitment on the Chinese Clinical Trial Registry (http://www.chictr.org.cn/showproj.aspx?proj=55262 No.ChiCTR2000034313). We will conduct the principles of the Declaration of Helsinki (2004 version) during the trial. The study protocol also has been approved by the Ethics Committee of the First Affiliated Hospital of Zhejiang Chinese Medical University before recruitment (Approval number: 2019-KL-108–01). This study is a randomized, placebo-controlled, blind trial. The study flowchat was shown in Figure 1.

### 2.2 Recruitment

This study will be conducted in the The First Affiliated Hospital of Zhejiang Chinese Medical University (Hangzhou, China). Participants will be recruited through a recommendation by the mammary surgeon. Before enrollment, participants will be informed detail information about the trial, including its purpose, processing, scheduling, and possible risks and benefits. All participants should sign the informed consents.

### 2.3 Inclusion criteria

Female patients with breast cancer aged 40 to 60 years old with pathologically confirmed breast cancer patients who met the CLINICAL practice guidelines for NCCN breast cancer (2018.v1); KPS score no less than 60 points, WBC no less than 4.0 × 10^9^ before treatment, normal liver and renal function, expected survival above three months; All were pathologically confirmed breast cancer patients who met the CLINICAL practice guidelines for NCCN breast cancer (2018.v1) after modified radical mastectomy and received chemotherapy with AC regimen (adriamycin + cyclophosphamide) or TAC regimen (docetaxel + adriamycin + cyclophosphamide) for the first time. All the selected patients have signed informed consent and are willing to accept the treatment of this program.

### 2.4 Exclusion criteria

Patients who are participating or were participated in other clinical trials within 1 month or are unable to provide written consent will be excluded from the trial. Patients diagnosed with primary or secondary cardiovascular, cerebrovascular, pulmonary, renal, endocrine, nerve, and hematology diseases will also be excluded from the study.

### 2.5 Sample size estimation

PASS software was used to estimate the sample size. In this study. According to the literature and our preliminary studies,^[13,14]^ the effective rates of the control group and the experimental group were respectively 53% and 75%. The two groups were distributed in a 1:1 ratio under the conditions of α = 0.05 and test efficiency, 1-β = 0.8. At a result, the sample size of each group was calculated to be at least 71 people. Considering the existence of factors such as shedding, the samples were expanded by 12% on the premise of ensuring the minimum sample size, and finally the samples were expanded to 80 cases in each of the two groups, a total of 160 cases.

### 2.6 Study drug

The experimental medicine is Shenhuang Plaster, consisting of *Radix et Rhizoma Rhei* (Rhubarb Root and Rhizome, Dahuang), *Radix Salviae Miltiorrhizae* (danshen root, Danshen), *Radix Ginseng* (Ginseng, Renshen), *Cortex Magnoliae Officinalis* (Officinal magnolia bark, Houpu), *Fructus Aurantii Immaturus* (Immature Orange Fruit, Zhishi), *Fructus Evodiae* (Medicinal Evodia Fruit, Wuzhuyu) and *Flos Caryophylli* (Clove, Dingxiang). The Shenhuang Plaster and the placebo will be produced by Chinese Herbal Pieces Co., Ltd. of Zhejiang Chinese Medical University. The manufacturing procedures are as follows: According to the formula ratio of Shenhuang Plaster (*Radix et Rhizoma Rhei*, *Radix Salviae Miltiorrhizae*, *Radix Ginseng*, *Cortex Magnoliae Officinalis*, *Fructus Aurantii Immaturus*, *Fructus Evodiae* and *Flos Caryophylli* (20: 20: 20: 7: 7: 3: 3)), each herb will be mixed with eight times amount of 60% ethanol and refluxed twice at 80°C water bath for 1 h each,filter and combine the filtrate. Add eight times the amount of water to the filtered residue, continue to boil and extract for 2 times, 1 h each time, filter and combine the filtrate. Mix the filtrates to a proper concentrate plaster and store in the 4 degree refrigerator.

### 2.7 Randomization and blinding

Stratified block randomization will be conducted by the Zhejiang Hospital of Traditional Chinese Medicine with their online central randomization system. The recruitment process will be overseen by a Clinical supervisor. Each patient will be given a unique ID through a web interface provided by Clinical Data Centre of Zhejiang Hospital of Traditional Chinese Medicine. Patients will be randomly allocated to the experimental group and the control group at a ratio of 1:1 according to the ID given by the central randomization system. Each trial drug will also be labeled with a unique number and dispatched with the online system at each visit. All experimental plaster and corresponding placebo plaster are consistent in appearance and taste. The grouping information of each participant will be concealed to all research personnel and data analyzers until the end of trial. Unblinding will be permitted when serious adverse events occur.

### 2.8 Interventions

This study will compare the experiment group with the control group. Patients meeting the inclusion criteria will be allocated to the experimental group or control group at a ratio of 1:1. Patients in the experimental group will receive the SHP application on CV8. Patients in the control group will receive the placebo plaster application on CV8. All patients of two groups will be treated with acupoint application once per day from the day before chemotherapy and will last 3 days. All plasters will be taken back by the experimenter to the research center after application.

The standard treatment procedures are as follows: Ensure the CV8, disinfect the application site with iodophor, allow to dry then stick the SHP or placebo plaster on the CV8 at about 8am. Each application lasts 10 hours. If patients have obviously burning sensation and severe itching, or skin redness, swelling, blisters and other discomfort, the plaster would be immediately removed the plaster and deal with corresponding treatment.

## 3 Data collection and management

### 3.1 Evaluation

#### 3.1.1 Primary outcome

The primary outcome will be the form of faces every day. The BSFS (Bristol Stool Form Scale)^[15]^ will be used to assess patients’ face form before, during and after the intervention. A higher BSF score indicates soft feces: 1 to 2 points indicate constipation, 3 to 4 points indicate normal feces, and 5 to 7 points indicate diarrhea.

#### 3.1.2 Second outcome

Secondary outcome measurements include the symptom score of TCM, the changes of fecal bacteria and metabolites, serum motilin, gastrin and ghrelin levels. The symptom of TCM will be assessed every day during the intervention, and other outcomes will be assessed before and after the treatment.

#### 3.1.3 Adverse events

All adverse events will be documented during the intervention. If any adverse event occurs, it will be immediately reported and the participant will be received the corresponding treatment. We will analyze the causality to determine the severity and the relationship between the adverse events. Serious adverse events determined to be drug-induced will be reported to the ethical committee timely and discuss whether we should discontinue or modify the criteria. The trial will be allowed the unblinding after obtaining the consent of the main investigator.

#### 3.1.4 Statistical analysis

The clinical data will be managed and analyzed by Clinical Data Center of Zhejiang Hospital of Traditional Chinese Medicine. Personal information about potential and enrolled participants will be collected by researchers and will updated in the Chinese Clinical Trial Registry before and during the trial. SPSS V.21.0 will be used for statistical analysis. SPSS software (Version 19.0) will be used for statistical analysis. The continuous data will be expressed as the mean ± standard deviation (SD). The categorical data will be expressed as numbers (percentages) or medians. If the P value less than 0.05 will be concerned that there is statistical significance.

## 4 Discussion

Treatment of breast cancer with chemotherapy is remarkably effective, but it is associated with side effects. The disruption in the function of the intestine is a common clinically side effect. Chemotherapy-induced gastrointestinal toxicity (CIGT) is associated with a variety of clinical complications, including anorexia, nausea, vomiting, diarrhea, constipation. CIGT affected more than half of cancer which will lead to the break and/or reduction of treatments patient, and has huge clinical and economic implications. Patients who experience severe CIGT have twice the infection risk leading to a fourfold higher chance of death and threefold longer hospital stays, compromising survival and creating a burden on patients’ quality of life.^[11]^ CIGT is considered to be an important factor affecting the recovery of cancer patients with chemotherapy treatment. But current evidences do not support widespread implementation of probiotics for CIGT.^[16,17]^ Acupoint therapy is one of the therapeutic methods in TCM based on acupoints and meridians theory, including acupuncture, electroacupuncture, moxibustion, acupoint application, acupoint injection, and catgut embedding. Acupoint application is an external treatment based on the theory of TCM. It applies Chinese herbal medicines to the skin at corresponding acupoints and has superior efficacy and fewer side effects than using Western medicine alone.^[18]^ Our previous studies observed that SHP can improve gastrointestinal toxicity, intestinal mucosal damage induced by chemotherapy.^[7,19,20]^ Each herb in SHP has been verified to regulate intestinal function, promote the increase of probiotics in the intestine, reduce the inflammation of the smooth muscle of the small intestine and accelerate the secretion of serum Ghrelin.^[21–24]^ Moreover, our another study suggests that the SHP was absorbed better through CV8 than non-acupoint.^[25]^ The efficacy and safety of the SHP on shenque to regulate gastrointestinal toxicity in breast cancer patients after chemotherapy will be observed in the current study.

In summary, this study is a large randomized, placebo-controlled, blind trial that aims to test the SHP application on CV8 will improve the gastrointestinal side effect after chemotherapy in breast cancer patients. If the results are positive, they will provide strong evidence of the contribution of acupoint therapy to the clinical outcomes in cancer patients.

### 4.1 Trial status

The protocol's version number and date are V1.0 and June 6, 2019. The first participants were recruited in December 1 2020 and will be finished approximately in June 30, 2021.

The recruitment is currently open. The data will be updated in the Chinese Clinical Trial Registry. (Supplimentary digital content appendices docx).

## Author contributions

RW and QS contributed conception and design of the study. XX, QG, RW, and JX, and QS organized the investigation. YS performed the statistical analysis and prepared the figures and tables. QS and XX (Xiaohong Xu) contributed supervision. RW and YS wrote the first draft of the manuscript. QS and XX (Xiaohong Xu) wrote the sections of the manuscript. All authors contributed to manuscript revision, read, and approved the submitted version.

**Conceptualization:** Rongyun Wang, Yanan Shi, Qiuhua Sun, Xiaohong Xu.

**Data curation:** Rongyun Wang, Yanan Shi, Qinling Ge.

**Methodology:** Rongyun Wang, Qiuhua Sun, Xiaohong Xu.

**Project administration:** Qiuhua Sun.

**Resources:** Xiaohong Xie, Qinling Ge, Jingming Xu, Xiaohong Xu.

**Software:** Jingming Xu.

**Supervision:** Xiaohong Xie, Qiuhua Sun, Xiaohong Xu.

**Writing – original draft:** Rongyun Wang, Yanan Shi.

## Supplementary Material

Supplemental Digital Content
