# Supplementary material for: Use of Shenhuang paste on Shenque point improves chemotherapy induced gastrointestinal toxicity in breast cancer: A protocol for randomized controlled trial
Source: Medicine (Baltimore). 2021 Apr 16;100(15):e25097. doi: 10.1097/MD.0000000000025097 (PMC8052020; doi:10.1097/MD.0000000000025097)
Supplement: Supplemental Digital Content [file medi-100-e25097-s001.docx]

**Appendices**

**[Informed](javascript:;)** **[Consent](javascript:;)**

参黄膏贴敷神阙穴治疗乳腺癌化疗相关性胃肠功能紊乱的研究

知情同意书·知情告知页

版本号：V1.0

版本日期: 2019.6.25

亲爱的患者：

我们将邀请您参加一项参黄膏贴敷神阙穴治疗乳腺癌化疗相关性胃肠功能紊乱的研究,以观察他们对于化疗相关性胃肠功能紊乱的疗效和安全性。治疗药物是通过穴位敷贴的方式给药。

在您决定是否参加这项研究之前，请尽可能仔细阅读以下内容，它可以帮助您了解该项研究以及为何要进行这项研究，研究的程序和期限，参加研究后可能给您带来的益处、风险和不适。如果您愿意，您也可以和您的亲属、朋友一起讨论，或者请您的医生给予解释，帮助您做出决定。

研究介绍

**一、研究背景和研究目的**

化疗相关性胃肠道功能紊乱（Chemotherapy-induced Gastrointestinal Toxicity，CIGT）是临床肿瘤化疗常见并发症，常表现为恶心呕吐、腹痛腹胀、食欲减退、腹泻便秘及烦躁焦虑等症状，轻者影响患者生活质量及对化疗的依从性，重者导致化疗终止，甚至诱发肿瘤复发。据统计，超过50%肿瘤化疗患者发生严重CIGT 时全身感染风险提高2 倍，住院时间延长3倍，死亡风险提高4倍。即使化疗结束 CIGT 的影响也可持续10年。目前CIGT被认为是影响肿瘤化疗患者 康复的重要因素和核心环节。本项目采用双盲随机对照研究，将名老中医经验方参黄膏贴敷于乳腺癌化疗后肠道功能紊乱患者脐部，观察患者临床效应、血清胃肠道激素和粪细菌菌群结构的改变。评价参黄膏敷脐在促 进乳腺癌化疗后胃肠功能功能紊乱的有效性及安全性，并在此基础上制定参黄膏剂型规范、参黄膏敷脐疗法治疗乳腺癌化疗后胃肠功能紊乱的外治技术方案应用规范，为后续新药开发奠定基础。

该研究目的：①明确参黄膏贴敷神阙穴治疗乳腺癌化疗相关性胃肠道功能紊乱的临床效果；②形成规范的参黄膏贴敷神阙穴干预乳腺癌化疗后相关性胃肠功能紊乱的干预方案；③探讨参黄散贴敷神阙穴治疗乳腺癌化疗相关性胃肠道功能紊乱的效应机制；④若研究结果疗效确切，进一步对参黄膏制剂进行开发和推广应用，争取取得更大的社会效益和经济效益。

本项研究已经得到国家药品监督管理局批准。伦理委员会已经审议此项研究是遵从赫尔辛基宣言原则，符合医疗道德的。

**二、哪些人不宜参加研究**

(1)合并肺心病、呼吸衰竭的患者；

(2)妊娠和哺乳期妇女，及已知对本试验用药成分过敏者；

(3)合并有甲状腺功能亢进，心脑血管、肝肾和造血系统等严重原发性疾病、精神病患者。

⑷正在参加其它临床研究者。

**三、如果参加研究将需要做什么**

1. 在您入选研究前，您将接受以下检查以确定您是否可以参加研究：

医生将询问、记录您的病史，对您进行体格检查。

2. 若您已完成以上检查，将按以下步骤进行研究（按随访时点详细陈述治疗及各检查项目）

研究开始将根据计算机提供的随机数字，决定您接受试验组或对照组方案,A）对照组：对照剂敷贴；B）试验组：参黄膏敷贴。参加这项研究的患者分别有50%的可能性被分入这两个不同的组别。您和您的医生都无法事先知道和选择任何一种干预措施。治疗观察将持续4天。

参黄膏及参黄膏对照剂膏剂贴敷：于化疗当天开始使用，每日1次，每次贴敷20个小时，于当日上午10点左右贴敷，次日上午6点左右去除，连续使用4天。

贴敷方法:取神阙穴，用生理盐水棉球以肚脐为中心涂擦腹部皮肤两遍(范围约16cm×17cm)，然后用干纱布擦干皮肤。取参黄膏制剂1份，将无纺胶布与PCV分离，以肚脐(神厥穴) 为中心贴于腹部，粘贴牢固。取下敷贴后，用生理盐水棉球擦拭皮肤残留药膏，并记录贴敷过程中出现的是否出现过敏等不良反应，预防不良事件发生。

化疗前与化疗第3天：收集粪便，采取静脉血；

化疗前、化疗后1天、3天：评价临床症状；

血常规，化疗前、化疗第1天，第3天

肝、肾功能检查，化疗前天，化疗第3天

可能的不良事件，随时观察

3. 需要您配合的其他事项

您需要按医生指导进行穴位贴敷，并告知一声您正在服用的其它药物，包括您有其它合并疾病须继续服用的药物。

在研究期间您不能使用治疗化疗所致胃肠功能紊乱的其它药物。如您需要进行其它治疗，请事先与您的医生取得联系。

**四、参加研究可能的受益**

您和社会将可能从本项研究中受益。此种受益包括您的病情有可能获得改善，以及本项研究可能帮助开发出一种新治疗方法，以用于患有相似病情的其他病人。

**五、参加研究可能的不良反应、风险和不适、不方便**

您在参加此项研究的过程中，可能会出现皮肤发红，皮肤瘙痒，如果在研究期间您出现任何不适，或病情发生新的变化，或任何意外情况，不管是否与研究有关，均应及时通知您的医生，他/她将对此作出判断并给与适当的医疗处理。

医生和课题组将尽全力预防和治疗由于本研究可能带来的伤害。如果在临床研究中出现不良事件，科研项目所属政府管理部门及医院伦理委员会将会鉴定其是否与本研究有关。研究者/课题组将对与研究相关的损害提供治疗的费用及相应的经济补偿。

此外，（研究干预）可能出现无效的情况，以及因治疗无效或者因合并其他疾病等原因而导致病情继续发展。在研究期间，如果医生发现本项研究所采取的（研究干预）措施无效，将会中止研究，改用其他可能有效的治疗措施。

**六、有关费用**

如果发生与研究相关的损害，课题组将支付您的医疗费用。如果在临床试验中出现不良事件，医学专家委员会将会鉴定其是否与本研究或基础治疗药物有关。课题组将按照我国《药物临床试验质量管理规范》的规定对与试验相关的损害提供治疗的费用及相应的经济补偿）。

对于您同时合并的其他疾病所需的治疗和检查，将不在免费的范围之内。

**七、个人信息是保密的吗？**

您的医疗记录（研究病历/CRF、化验单等）将完整地保存在医院，医生会将化验检查结果记录在您的门诊病历上。研究者或课题组成员、伦理委员会和课题所属政府部门将被允许查阅您的医疗记录。任何有关本项研究结果的公开报告将不会披露您的个人身份。我们将在法律允许的范围内，尽一切努力保护您个人医疗资料的隐私。

除本研究以外，有可能在今后的其他研究中会再次利用您的医疗记录和病理检查标本。您现在也可以声明拒绝除本研究外的其他研究利用您的医疗记录和病理标本。

**八、怎样获得更多的信息？**

您可以在任何时间提出有关本项研究的任何问题。您的医生将给您留下他/她的电话号码以便能回答您的问题。

如果您对参加研究有任何抱怨，请联系医院伦理委员会办公室。

如果在研究过程中有任何重要的新信息，可能影响您继续参加研究的意愿时，您的医生将会及时通知您。

**九、可以自愿选择参加研究和中途退出研究**

是否参加研究完全取决于您的自愿。您可以拒绝参加此项研究，或在研究过程中的任何时间退出本研究，这都不会影响您和医生间的关系，都不会影响对您的医疗或有其他方面利益的损失。

您的医生或研究者出于对您的最大利益考虑，可能会随时中止您参加本项研究。

您可以不参加本项研究，或中途选择退出研究。

如果您因为任何原因从研究中退出，您可能被询问有关您使用试验药物的情况。如果医生认为需要，您也可能被要求进行实验室检查和体格检查。这对保护您的健康十分有利。

**十、现在该做什么？**

是否参加本项研究由您自己决定。您可以和您的家人或者朋友讨论后再做出决定。

在您做出参加研究的决定前，请尽可能向您的医生询问有关问题，直至您对本项研究完全理解。

感谢您阅读以上材料。

如果您决定参加本项研究，请告诉您的医生或研究助理，他/她会为您安排一切有关研究的事务。

请您保留这份资料。

知情同意书·同意签字页

临床研究项目名称：参黄膏贴敷神阙穴治疗乳腺癌化疗相关性胃肠功能紊乱的研究

临床研究开展单位：浙江中医药大学附属第一医院

伦理审查批件号：

同意声明

我已经阅读了上述有关本研究的介绍，而且有机会就此项研究与医生讨论并提出问题。我提出的所有问题都得到了满意的答复。

我知道参加本研究可能产生的风险和受益。我知晓参加研究是自愿的，我确认已有充足时间对此进行考虑，而且明白：

Ⅰ我可以随时向医生咨询更多的信息。

Ⅱ我可以随时退出本研究，而不会受到歧视或报复，医疗待遇与权益不会受到影响。

我同样清楚，如果我中途退出研究，特别是由于药物的原因使我退出研究时，我若将病情变化告诉医生，完成相应的体格检查和理化检查，这将对我本人和整个研究十分有利。

如果因病情变化我需要采取任何其他的药物治疗，我会在事先征求医生的意见，或在事后如实告诉医生。

我同意伦理委员会或申办者代表及研究质量监察人员查阅我的研究资料。

我同意□ 或拒绝□ 除本研究以外的其他研究利用我的医疗记录和病理检查标本。

我将获得一份经过签名并注明日期的知情同意书副本。

最后，我决定同意参加本项研究。

受试者签名： ＿ ＿ ＿ ＿ 年 ＿ ＿ 月 ＿ ＿ 日

受试者联系电话： 手机号：

法定代理人签名（如有）： 日期： 年 月 日

我确认已向患者解释了本研究的详细情况，包括其权利以及可能的受益和风险，并给其一份签署过的知情同意书副本。

研究者签名： 日期：＿ ＿ ＿ ＿ 年 ＿ ＿ 月 ＿ ＿ 日

研究者工作电话： 手机号码：

**浙江中医药大学附属第一医院伦理委员会办公室联系电话：**

0571-87013311
